# Supplementary material for: The first complete mitochondrial genome of Diadema antillarum (Diadematoida, Diadematidae)
Source: GigaByte. 2022 Nov 22;2022:gigabyte73. doi: 10.46471/gigabyte.73 (PMC9693923; doi:10.46471/gigabyte.73)
Supplement: Translation

# Translation: El primer genoma mitocondrial completo de *Diadema antillarum* (Diadematoida, Diadematidae)

**Authors:** Audrey J. Majeske, Alejandro J. Mercado Capote, Aleksey Komissarov, Anna Bogdanova, Nikolaos V. Schizas, Stephanie O. Castro Márquez, Kenneth Hilkert, Walter Wolfsberger, Tarás K. Oleksyk

**Affiliations:** ^1^ University of Puerto Rico at Mayagüez, Department of Biology, Call Box 9000, Mayagüez, PR 00681, USA; ^2^ Oakland University, Department of Biological Sciences, 118 Library Drive, Rochester, MI 48309, USA; ^3^ Applied Genomics Laboratory, SCAMT Institute, ITMO University, Lomonosova 9 Str., Saint Petersburg 197101, Russia; ^4^ University of Puerto Rico at Mayagüez, Department of Marine Sciences, Call Box 9000, Mayagüez, PR 00681, USA; ^5^ Uzhhorod National University, Faculty of Biology, Universytets’ka St, 14, Uzhhorod, Zakarpattia Oblast 88000, Ukraine

## Abstract

El genoma mitocondrial del erizo de mar negro de espinas largas, *Diadema antillarum*, se secuenció utilizando la tecnología de secuenciación de nueva generación de Illumina. El mitogenoma completo tiene un tamaño de 15,708 pb, que contiene dos ARNr, 22 ARNt y 13 genes codificadores de proteínas, además de una región de control no codificante de 133 pb. La composición de nucleótidos es 18.37% G, 23.79% C, 26.84% A y 30.99% T. El sesgo A+T es del 57.84%. El análisis filogenético basado en 12 genomas mitocondriales completos de erizos de mar, incluyendo cuatro especies de la familia Diadematidae, apoya la monofilia familiar. Sin embargo, las dos especies de *Diadema* en este estudio, *D. antillarum* y *D. setosum* no fueron identificadas como taxones hermanos.

## Descripción de los datos

El erizo de mar negro de espinas largas, *Diadema antillarum* (Diadematoida, Diadematidae; NCBI:txid105358; urn:lsid:marinespecies.org:taxname:124332), es un invertebrado marino bentónico que habita en las aguas poco profundas del océano Atlántico y el mar Caribe. Es un herbívoro importante y una especie clave que ayuda a mantener saludables los sistemas de arrecifes de coral a lo largo de sus hábitats marinos costeros [1–9]. *Diadema antillarum* fue abundante a consecuencia del impacto humano en la sobreexplotación de peces herbívoros más grandes y la desaparición de vertebrados más grandes. Junto con otros peces herbívoros más pequeños, servían como los principales herbívoros manteniendo la salud del sistema de arrecifes dominado por corales, hasta mediados de la década de 1980 [9–11]. Después del histórico evento de mortandad de esta especie en 1983–84, que presumiblemente fue causado por un patógeno desconocido transmitido por el agua, se produjeron repetidos eventos de mortandad en la década de 1990 hasta la actualidad [12]. Estos eventos han diezmado y aniquilado poblaciones en algunas localidades del Caribe [13–15]. Los esfuerzos de monitoreo y recuperación han sido prometedores, pero es probable que ocurran más eventos de mortandad a medida que continúe el cambio climático global [16–18].

## Contexto

Al momento, solo se han descrito unos pocos genes en el genoma mitocondrial de *D. antillarum*, incluídas secuencias parciales de *COI, COII* y ATP6, así como secuencias ensambladas de *ATP8* y *ARNt*^*Lys*^ [19, 20]. Se han completado más de 40 genomas mitocondriales de diferentes especies de erizos de mar, incluyendo sólo tres especies dentro de la familia Diadematidae, que contienen 12 géneros en total. De estas especies, ambas especies del género *Echinothrix* tienen genomas mitocondriales completos además de una de las siete especies dentro del género *Diadema*, específicamente *D. setosum*. Aquí, reportamos el genoma mitocondrial completo de *D. antillarum* y exploramos la utilidad de los genomas mitocondriales completos para colocar a *D. antillarum* filogenéticamente en el árbol de Diadematidae.

## Métodos

### Recolección de muestras y extracción de ADN

Un erizo de mar adulto fue recolectado en Puerto Rico (18^°^20*′*35.2*′′*N 67^°^15*′*40.5*′′*W). Se extrajo una muestra que contenía celomocitos enteros del animal a través de la linterna de Aristóteles utilizando una aguja estéril de calibre 23 conectada a una jeringa de 5 ml. El animal fue fotografiado y regresado a su hábitat en el sitio de recolección. No se conservó como un espécimen de libre acceso para este estudio. La muestra se mantuvo en hielo durante su transporte a la Universidad de Puerto Rico Mayagüez (UPRM). El ADN total se extrajo de los celomocitos utilizando un kit de sangre y tejido DNeasy^®^ (Qiagen, Inc.) según las instrucciones de la compañía. La calidad y la concentración del ADN de la muestra se evaluaron utilizando un espectrofotómetro NanoDrop 2000 (ThermoFisher Scientific) antes del envío para su secuenciación en el laboratorio Psomagen (Macrogen USA) en Rockville, MD, EUA. La aprobación para la recolección de muestras se obtuvo del Departamento de Recursos Naturales y Ambientales de Puerto Rico (O-VS-PVS15-AG-00046-01082018).

### Secuenciación y análisis bioinformático

Para preparar la muestra de ADN para la secuenciación, se generó una biblioteca de secuencias utilizando un kit de preparación de biblioteca TruSeq^®^ DNA PCR Free (350) (Illumina, Inc.). La verificación de calidad y cuantificación de las bibliotecas se realizó utilizando un Bioanalizador. Luego la plataforma Illumina HiSeq 2500 generó lecturas de secuencia en pares con 151-pares de base (pb). El secuenciador produjo un total de 60,8 Gpb y 402, 874, 618 lecturas de extremo pareado, de las cuales el 88.21% tenía una puntuación de calidad de ≥Q30.

Los secuencias crudas candidatas para el genoma mitocondrial se extrajeron bioinformáticamente utilizando el software Cookiecutter2 del conjunto de datos completos producidos por Illumina que representan los genomas nuclear y mitocondrial [21, 22]. Las lecturas se recortaron utilizando un programa personalizado llamado v2trim [22].

Subsiguientemente, la lista de k-mers se formó en dos etapas. Primero, utilizamos el genoma de referencia más conocido de erizos de mar (*Strongylocentrotus purpuratus*, NC_001453.1 [23]) para ensamblar el borrador de contigs del ADNmt de *D. antillarum* utilizando el ensamblador del genoma SPAdes v3.15.4 (RRID: SCR_000131) [24]. Los contigs ensamblados fueron buscados en la interfaz web BLAST del Centro Nacional de Información Biotecnológica (NCBI, por sus siglas en inglés) [25] para encontrar la secuencia de referencia más cercana que coincidió con la especie *Echinothrix diadema*, con el número de acceso KX385836.1. En la segunda etapa, construímos la lista de k-mer usando ésta secuencia de referencia más cercana. Las secuencias extraídas se ensamblaron con SPAdes utilizando parámetros predeterminados. Para verificar la calidad del ensamblaje, se alinearon las secuencias extraídas al ensamblaje del ADNmt completo con BWA-MEM2 (RRID:SCR_022192) utilizando los parámetros predeterminados [26, 27]. La gráfica de cobertura se calculó con bedtools genomecov con parámetros –d y –split (BEDTools RRID:SCR_006646) [28]. Además, se generó una tabla de datos de cobertura por base con la herramientas de análisis de datos de NGS proporcionadas en el programa de software Unipro UGENE [29]. Se generó un gráfico de esta tabla de datos en Microsoft Excel (2019) presentada como Figura 1. Finalmente, el inicio del ensamblaje se rotó a la posición de tRNA-Phe. Después del ensamblaje, se realizó una anotación en línea utilizando el servidor web MITOS [30] seguida de una verificación manual de cada ARN y proteína predichos. Los resultados de esta anotación se utilizaron para generar un mapa mitocondrial en Geneious Prime v2022.1.1 (Figura 2) [31]. Las secuencias extraídas, los archivos de cobertura de bam y la lista de comandos reproducibles están disponibles en nuestro repositorio de GitHub [32].

**Figura 1.** Mapa de cobertura de ensamblaje del mitogenoma de *Diadema antillarum*. Se generó una tabla de datos de cobertura por base utilizando Unipro UGENE. Este gráfico se generó en Microsoft Excel (2019). El eje *X* muestra la posición de las bases de la secuencia del mitogenoma. El eje *Y* muestra la cobertura en esa base. El tamaño del mitogenoma es de 15.708 pb.

**Figura 2.** Mapa del genoma mitocondrial de *Diadema antillarum*. Las formas de flecha están coloreadas para indicar genes de ARNt (azul), genes de ARNr (rojo) y genes codificadores de proteínas (verde). La forma rectangular negra se refiere a la región de control no codificante o bucle D. Las direcciones de las flechas indican la dirección de transcripción en la hebra H (la flecha apunta a la derecha) y la hebra L (la flecha apunta a la izquierda). El mapa se generó en Geneious Prime v2022.1.1. La imagen animal es el espécimen utilizado para el muestreo en este estudio. Imagen tomada por Alejandro Mercado Capote.

### Análisis filogenético

La alineación de secuencias y el análisis filogenético se construyeron en MEGA v11.0.11 [33]. El análisis incluyó los genomas mitocondriales completos de 12 especies representativas de los órdenes Camarodonta (familias Temnopleuridae, Echinometridae y Parechinidae), Arbacioida (familia Arbaciidae), Temnopleuroida (familia Toxopneustidae), Cidaroida (familia Cidaridae), Echinoida (familia Strongylocentrotidae) y Diadematoida (familia Diadematidae), más el pepino de mar, *Apostichopus japonicus*. Se implementó MUSCLE como método de alineación utilizando los parámetros predeterminados en el programa MEGA11. Para generar una matriz de identidad de secuencia de las secuencias alineadas se utilizó otro editor de alineación de secuencias, BioEdit [34]. Realizamos un análisis IQ-TREE para determinar el modelo de sustitución más adecuado entre las secuencias del mitogenoma [35]. Se generó un árbol de máxima verosimilitud (ML, por sus siglas en inglés) con las secuencias de ADN de nucleótidos del mitogenoma de las 12 especies de erizo de mar y el pepino de mar incluí tiempo general reversible do como grupo externo. Para el análisis filogenético, elegimos un modelo completo de parámetros que resultó en ramas que contenían los mejores valores sustentados de “bootstrap” (de 500 iteraciones de “bootstrap”) para el árbol de consenso resultante. El árbol ML se generó utilizando un modelo de evolución Tamura-Nei [36]. Se utilizó una distribución gamma discreta para modelar las diferencias de tasa evolutiva entre las posiciones genómicas (cinco categorías (+*G*, parámetro = 0,3129)). Los árboles iniciales para la búsqueda heurística se obtuvieron automáticamente aplicando los algoritmos Neighbor-Join y BioNJ a una matriz de distancias por pares estimadas utilizando el modelo Tamura-Nei, y luego el árbol ML se generó seleccionando la topología con probabilidad logarítmica superior. Se realizaron alineamientos de secuencias adicionales y análisis filogenéticos en las secuencias disponibles para *Echinothrix* spp. y *Diadema* spp. para cada uno de los tres genes: ARN ribosómico 16S (secuencia parcial), la subunidad 1 de la citocromo oxidasa (CO1) (secuencia codificante parcial) y genes de ATPasa (incluyendo parte de la secuencia codificante del gen de la subunidad 6 de la ATP sintetasa, la secuencia parcial del gen tRNA-Lys, la secuencia codificante completa del gen ATP8 y parte de la secuencia codificante del gen ATP6). Para estos análisis adicionales, se generó una alineación MUSCLE utilizando los parámetros predeterminados en MEGA11. Además, se extrajeron las porciones del mitogenoma para *E. diadema* (KX385836), D. *setosum* (KX385835) y *D. antillarum* (este estudio) que correspondían a cada gen, generando una alineación inicial utilizando secuencias disponibles de los mismos géneros para cada uno de los tres genes. Se obtuvo un árbol de ML para cada gen utilizando un modelo “bootstraped” (500 iteraciones) de tiempo general reversible (GTR, por sus siglas en inglés) con tasas de sustitución invariantes (I) entre los nucleótidos. Los árboles iniciales se obtuvieron aplicando los algoritmos Neighbor-Join y BioNJ a una matriz de distancias por pares estimadas utilizando el modelo Tamura-Nei, y luego se generó el árbol ML seleccionando la topología con un valor de probabilidad logarítmica superior.

## Validación de datos y control de calidad

### Características del mitogenoma

El mitogenoma circular de *D. antillarum* tiene un tamaño de 15,708 pb, que comprende dos genes de ARNr, 22 genes de ARNt, una región de control no codificante y 13 genes codificadores de proteínas que son comunes en otros equinodermos, así como el orden de los genes [37–39]. La mayoría de los genes están codificados en la cadena H, excepto un gen codificador de proteínas, *ND6*, y cinco genes de ARNt, incluidos el ARNt^*Gln*^, el ARNt^*Ala*^, el ARNt^*V al*^, el ARNt^*Asp*^ y *elARNt*^*Ser*^, que están codificados en la cadena L [40]. La mayoría de los genes codificadores de proteínas utilizan el codón de inicio ATG, con la excepción de *ATP8*, que comienza con GTG. La longitud de los genes de ARNr es de 896 pb para el ARNr 12S y de 1,555 pb para el ARNr 16S. La región de control es de 133 pb y contiene la repetición G típica que se encuentra en otros equinodermos. Esta región no codificante se encuentra en las bases en las posiciones 1,111 a 1,243, y se encuentra entre los genes tRNA^*Thr*^ y *tRNA*^*Pro*^.

La composición de los nucleótidos para el mitogenoma de *D. antillarum* se calculó en MEGA11 como 1.37% G, 23.79% C, 26.84% A y 30.99% T. El sesgo A + T también se calculó en MEGA, para D. *antillarum* es de 57.84%, ligeramente inferior, pero comparable con el de *D. setosum*, que es de 58.19%. En comparación con las otras dos especies de la familia *Echinodrix* a la que *D. antillarum pertenece*, el sesgo A + T en el mitogenoma de *D. antillarum* es ligeramente superior al de *E. diadema* (57.61%) y *E. calamaris* (56.43%). No obstante, cuando se compara con especies en diferentes órdenes, el sesgo mitocondrial A + T de *D. antillarum* suele ser menor que otras especies en los siguientes órdenes: Cidaroida (*Stylocidaris reini*, 59.93%; *Prionocidaris baculosa*, 59.14%; *Eucidaris tribuloides*, 59.70%), Camarodonta (*Echinometra mathaei*, 59.21%; *Heterocentrotus mamillatus*, 58.91%; *Heliocidaris crassispina*, 58,89%), y Echinoida (*Strongylocentrotus droebachiensis*, 58.96%; *S. intermedius*, 58.92%; *S. purpuratus*, 58.98%).

**Figura 3.** El análisis filogenético de los genomas mitocondriales indica que *Diadema antillarum* está más estrechamente relacionada con *Echinothrix diadema* que con *D. setosum*. Un árbol de consenso de máxima verosimilitud de secuencias que representan 12 especies de erizos de mar y un pepino de mar (grupo externo). Los números de acceso del Genbank para los taxones en el árbol incluyen: *H. crassispina* (KC479025.1 [43]), S. *purpuratus* (NC_001453.1 [23, 44, 45]), *L. variegatus* (NC_037785.1 [37]), *T. gratilla* (KY268294.1 [46]), *P. lividus* (J04815.1 [47]), S. *sphaeroides* (KU302103.1 [41]), A. *lixula* (X80396.1 [39]), E. *calamaris* (NC_050274.1 [48]), *D. setosum* (KX385835.1 [49]), E. *diadema* (KX385836.1 [50]), E. *tribuloides* (MH614962.1 [51]) y *A. japonicus* (NC_012616 [52]). Las longitudes de las ramas se igualan y no incluyen los tiempos de divergencia evolutiva. Los valores de soporte de Bootstrap se incluyen en las ramas del árbol.

**Figura 4.** Análisis filogenético de secuencias de ARN ribosómico 16S para *Diadema y Echinothrix* spp. Un árbol de consenso de máxima verosimilitud de secuencias genéticas parciales para 19 secuencias de *Diadema y Echinothrix* spp. más *Eucidaris tribuloides* como un grupo externo. Se extrajo una sección del mitogenoma para *E. diadema* (KX385836), *D. setosum* (KX385835), *D. antillarum* (10.02.2022) y *E. tribuloides* (ETRI 00-1) que correspondía al gen 16S generando un alineamiento inicial. Para ello, las secuencias específicas del gen E. *calamaris* 16S se alinearon con todo el mitogenoma de *E. tribuloides* y *E. diadema*. Luego, las secuencias específicas del gen *D. setosum y D. savignyi* para 16S se alinearon con todo el mitogenoma de *E. tribuloides* y *D. antillarum*. Por último, las secuencias específicas del gen D. *setosum* enumeradas en el árbol se alinearon con todo el mitogenoma de *E. tribuloides* y *D. setosum*. El resto del mitogenoma de cada especie se recortó para reflejar solo la porción perteneciente a la secuencia del gen 16S. Por lo tanto, los taxones en el árbol llamado Diadema antillarum mtDNA 10.02.2022, Echinothrix diadema KX385836=NC 033523, Diadema setosum KX38535=NC 033522 y Eucidaris tribuloides aislado ETRI 00-1 MH614961=NC 050252 incluir solo las secuencias del gen 16S. Las longitudes de las ramas se igualan y no incluyen los tiempos de divergencia evolutiva. Los valores de soporte de Bootstrap se incluyen en las ramas del árbol.

### Árbol filogenético

El árbol consenso de ML con “bootstrap” se muestra en la Figura 3. Esto se dedujo de 500 réplicas a través de la agrupación de los taxones asociados [41]. Los resultados del análisis IQ-TREE indicaron que el mejor modelo de sustitución fue el modelo más completo probado, el modelo GTR+F+R2 (modelo reversible entiempo general con tasas desiguales y frecuencia base desigual con modelo de heterogeneidad de tasas FreeRate) [42]. Los resultados de esta prueba incluyeron un árbol de ML que carecía de un análisis “bootstrap”, que está disponible en GitHub [32]. Además, se generó un árbol de ML en el programa MEGA11 utilizando el modelo de evolución GTR sugerido con tasas de sustitución invariantes (o desiguales) entre sitios. Los dos árboles de consenso ML resultantes de MEGA11, más el árbol ML generado por el análisis IQ-TREE, tenían topologías idénticas con respecto a las especies dentro de la familia Diadematidae. De los dos árboles ML “bootstrap” producidos en MEGA, el árbol presentado en este estudio tendió a mostrar valores de “bootstrap” más altos para todas las ramas dentro del árbol.

La topología del árbol mostrada en este estudio indicó que *D. antillarum* estaba más estrechamente relacionado con *E. diadema* que con *D. setosum*. El siguiente taxón más estrechamente relacionado con estos tres fue *E. calamaris*. Para *E. diadema*, *E. calamaris* y *D. setosum*, el tamaño de estos genomas mitocondriales es de 15,712 pb, 15,716 pb y 15,708 pb, respectivamente. Un estudio previo reportó el mitogenoma completo de *D. setosum* e incluyó un árbol filogenético ML con un clado hermano altamente sustentado (valor de “bootstrap” de 100) que contiene dos taxones: *E. diadema* y *D. setosum* [49]. Además, los resultados de nuestra matriz de identidad de secuencia de mitogenomas entre *D. antillarum* y *E. diadema* fueron 96.7% idénticos, mientras que *D. antillarum* vs. D. *setosum* o *D. antillarum* vs. E. *calamaris* tuvieron 86.3% y 80% de identidad, respectivamente. Además, los mitogenomas entre *E. diadema* y *D. setosum* fueron 86.2% idénticos en secuencia. Esto podría sugerir que la mayor similitud general entre *D. antillarum* y *E. diadema*, en comparación con *D. setosum*, podría estar asociada con el mayor sesgo A + T en el mitogenoma. Sin embargo, las secuencias del mitogenoma de *E. diadema* y *D. setosum* se generaron a partir de especímenes recolectados en el Mar del Sur de China por el mismo grupo de investigación [49, 50]. Dado que hay otras especies de *Diadema* en el Mar del Sur de China que son morfológicamente similares a *E. diadema* (incluyendo *D. setosum* y *D. savignyi*), el mitogenoma reportado de *E. diadema* puede haber sido muestreado de una especie de *Diadema*, es decir, *D. savignyi*. Desafortunadamente, el mitogenoma de *D. savignyi* no se ha completado o no está disponible. No obstante, para proporcionar evidencia de esta afirmación, realizamos un análisis de secuencia para tres genes adicionales (*16S, ATPasa* y *CO1*) para los datos disponibles de especies de los géneros *Echinothrix* y *Diadema*. Los resultados de las topologías de árbol para los tres genes indican que las secuencias genéticas específicas extraídas del mitogenoma de *E. diadema* están más estrechamente relacionadas con la secuencia genómica obtenida de *Diadema sp*. en lugar de los de *Echinothrix* sp. (Figuras 4, 5 y 6). En particular, las secuencias genéticas específicas extraídas del mitogenoma de *E. diadema* se localizaron como un clado hermano con *D. savignyi* (ver Figuras 4–6), que se colocó junto a un grupo más grande de clados que incluía *D. africanum* (ver Figura 4), *D. antillarum* (ver Figuras 4–6) *D. mexicanum* (véase la Figura 4) y *D. setosum* (véase la Figuras 4–6). Esto se relacionó más lejanamente con las agrupaciones de clados que incluían *E. diadema* (ver Figura 4) y *E. calamaris* (ver Figuras 4–6).

**Figura 5.** Análisis filogenético de los genes ATPasa para *Diadema y Echinothrix* spp. En el análisis se incluyeron el gen de la subunidad 6 de la ATP sintasa (secuencia codificante parcial) y el gen de la subunidad 8 de la ATP sintasa (secuencia codificante completa), así como el gen tRNA-Lys (secuencia parcial), que se incluyó en algunos de los taxones. Se muestra un árbol de consenso de máxima verosimilitud para 16 secuencias de *Diadema y Echinothrix* spp. más *Eucidaris tribuloides* como un grupo externo. Se extrajo una sección del mitogenoma para *E. diadema* (KX385836), *D. setosum* (KX385835), *E. calamaris* (KNZ-KUC07), *D. antillarum* (10.02.2022) y *E. tribuloides* (ETRI 00-1) que correspondía al gen específico generando un alineamiento inicial. Para hacer esto, las secuencias específicas del gen E. calamaris enumeradas en el árbol se alinearon con los mitogenomas completos de *E. tribuloides*, *E. diadema* y *E. calamaris*. Luego, las secuencias específicas del gen en *D. antillarum* enumeradas en el árbol se alinearon con todo el mitogenoma de *E. tribuloides* y *D. antillarum*. Por último, las secuencias específicas del gen *D. setosum* enumeradas en el árbol se alinearon con todo el mitogenoma de *E. tribuloides* y *D. setosum*. El resto del mitogenoma de cada especie se recortó para reflejar solo la porción perteneciente a las secuencias del gen ATPasa. Así, los taxones del árbol denominados *Diadema antillarum* mtDNA 10.02.2022, *Echinothrix diadema* KX385836=NC 033523, *Diadema setosum* KX38535=NC 033522, *Echinothrix calamaris* KNZ-KUC07 MK609484=NC050274 y Eucidaris tribuloides aislado ETRI 00-1 MH614961=NC 050252 incluir únicamente las secuencias del gen ATPasa. Las longitudes de las ramas se igualan y no incluyen los tiempos de divergencia evolutiva. Los valores de soporte de Bootstrap se incluyen en las ramas del árbol.

**Figura 6.** Análisis filogenético de los genes de la citocromo oxidasa 1 (*CO1*) para *Diadema y Echinothrix* spp. Se muestra un árbol de consenso de máxima verosimilitud para 15 secuencias de codificación parcial de *Diadema y Echinothrix* spp. más *Eucidaris tribuloides* como un grupo externo. Se extrajo una sección del mitogenoma para *E. diadema* (KX385836), *E. calamaris* (KNZ-KUC07), *D. antillarum* (10.02.2022) y *E. tribuloides* (ETRI 00-1) que correspondía al gen *CO1* generando un alineamiento inicial. Para hacer esto, las secuencias específicas del gen *E. calamaris* enumeradas en el árbol se alinearon con todo el mitogenoma de *E. tribuloides*, *E. diadema* y *E. calamaris*. Por separado, las secuencias específicas del gen *D. antillarum* enumeradas en el árbol se alinearon con todo el mitogenoma de *E. tribuloides* y *D. antillarum*. El resto del mitogenoma de cada especie se recortó para reflejar solo la porción perteneciente a la secuencia del gen *CO1*. Así, los taxones del árbol denominado *Diadema antillarum* mtDNA 10.02.2022, *Echinothrix calamaris* KNZ-KUC07 MK609484=NC050274, Echinothrix diadema KX385836=NC 033523 y *Eucidaris tribuloides* aislado ETRI 00-1 MH614961=NC 050252 incluir únicamente las secuencias del gen *CO1*. Las longitudes de las ramas se igualan y no incluyen los tiempos de divergencia evolutiva. Los valores de soporte de Bootstrap se incluyen en las ramas del árbol.

 Además, es importante tener en cuenta que los rangos de hábitat de *E. diadema* y *D. setosum* se encuentran en la región del Indo-Pacífico, que es distinta del rango de hábitat de *D. antillarum* que se encuentra típicamente en el Océano Atlántico occidental, el Mar Caribe, las costas tropicales de América del Sur hasta Brasil y desde Bermudas hasta Florida. Una nueva subespecie de *D. antillarum*, llamada *D. antillarum ascensionis*, ha sido identificada en el Atlántico oriental, que es similar a la especie del Atlántico medio de D. *antillarum* [53]. Esta nueva subespecie también es similar a *D. africanum* que se encuentra en las islas atlánticas orientales, desde las islas Madeira hasta el Golfo de Guinea, incluyendo Salvage, Canarias, Cabo Verde [54] y Santo Tomé y las islas Sâo Sâo [19]. A pesar de que *D. antillarum* ha estado repoblando áreas que han extendido su área de distribución, no tenemos conocimiento de ninguna migración de D. *africanum* tan al oeste como Puerto Rico, en el Mar Caribe. Además, al identificar el espécimen antes de la recolección para este estudio, carecía de los iridóforos, típicos de *D. africanum*, que son visibles a la luz del sol. Si bien el análisis de la secuencia ATPasa presentado en este estudio refleja que hay similitudes en estas secuenciasentre *D. antillarum*, *D. africanum y D. mexicanum*, la secuencia del gen *ATPasa* que se extrajo del mitogenoma utilizado en este análisis se parece más a la de otro *D. antillarum* (ver Figura 4). Por lo tanto, creemos que las especies utilizadas en este estudio fueron identificadas correctamente. Sin embargo, a medida que se disponga de secuencias adicionales del mitogenoma, el árbol filogenético que describe las relaciones entre géneros dentro de Diadematidae debería volverse más completo.

## Potencial de reutilización

Estos resultados pueden proveer una idea de los eventos de dispersión y especiación de la familia Diadematidae. Sin embargo, se necesitan más datos de otros géneros para llegar a más conclusiones sobre la evolución y adaptación de este linaje familiar. La secuencia del mitogenoma producida aquí puede servir como secuencia de referencia para esta especie de erizo de mar. Además, las secuencias nucleares generadas en este proyecto se incluirán en un estudio más amplio para ensamblar el genoma completo de esta especie. Con la importancia de *D. antillarum* para mantener la salud y la estructura actual del resto de las costas dominadas por corales frente al cambio climático global, será importante incluir la genética de esta especie en futuros estudios de conservación y genética de poblaciones.

## Data Availability

La secuencia del genoma mitocondrial ha sido depositada en DDBJ/ENA/GenBank bajo el número de acceso ON725136. Los datos están vinculados a los números NCBI BioProject y BioSample PRJNA839760 y SAMN28553754, respectivamente. Todos los archivos intermedios están disponibles en el repositorio GigaDB [55].

## Declaraciones

### Lista de abreviaturas

PA: par de bases; Gbp: pares gigabase; GTR: tiempo general reversible; ML: máxima verosimilitud.

### Aprobación ética

La aprobación para la recolección de muestras de erizo de mar se obtuvo del Departamento de Recursos Naturales y Ambientales de Puerto Rico (O-VS-PVS15-AG-00046-01082018).

### Consentimiento para la publicación

No aplica.

### Financiación

El financiamiento para el proyecto fue proporcionado por fondos iniciales otorgados a Tarás Oleksyk por la Universidad de Oakland.

## References

[1] LiddellWD, OhlhorstSL. Changes in benthic community composition following the mass mortality of Diadema at Jamaica. J. Exp. Mar. Biol. Ecol., 1986; 95(3): 271–278.

[2] HughesTP, ReedDC, BoyleM-J. Herbivory on coral reefs: Community structure following mass mortalities of sea urchins. J. Exp. Mar. Biol. Ecol., 1987; 113: 39–59.

[3] CarpenterRC. Mass mortality of a Caribbean sea urchin: Immediate effects on community metabolism and other herbivores. Proc. Natl. Acad. Sci. USA, 1988; 85: 511–514.1659390710.1073/pnas.85.2.511PMC279580

[4] CarpenterRC. Mass mortality of *Diadema antillarum*—I. Long-term effects on sea urchin population dynamics and coral reef algal communities. Mar. Biol., 1990; 104: 67–77.

[5] HughesTP. Catastrophes, phase shifts, and large-scale degradation of a Caribbean coral reef. Science, 1994; 265: 1547–1551.1780153010.1126/science.265.5178.1547

[6] Ferrari LegorretaR. Building resilience of Caribbean coral reefs to macroalgal phase shifts: Identifying key habitat features. PhD Thesis, School of Biological Sciences, The University of Queensland, Australia. 2012; https://espace.library.uq.edu.au/view/UQ:298060.

[7] Vega ThurberR, BurkepileDE, CorreaAMS Macroalgae decrease growth and alter microbial community structure of the reef-building coral, *Porites astreoides*. PLoS One, 2012; 7(9): e44246.2295705510.1371/journal.pone.0044246PMC3434190

[8] BurkepileDE, AllgeierJE, ShantzAA Nutrient supply from fishes facilitates macroalgae and suppresses corals in a Caribbean coral reef ecosystem. Sci. Rep., 2013; 3: 1493.2351221610.1038/srep01493PMC6504825

[9] SteneckRS. Sea urchins as drivers of shallow water benthic community structure. In: LawrenceJM (ed.), Sea Urchins: Biology and Ecology. San Diego, CA: Academic Press, 2020; pp. 195–212.

[10] JacksonJBC. What was natural in the coastal oceans? Proc. Natl. Acad. Sci. USA, 2001; 98(10): 5411–5418.1134428710.1073/pnas.091092898PMC33227

[11] PandolfiJM, BradburyRH, SalaE Global trajectories of the long-term decline of coral reef ecosystems. Science, 2003; 301(5635): 955–958.1292029610.1126/science.1085706

[12] Diadema Response Network. https://www.agrra.org/sea-urchin-die-off/. Accessed 24 May 2022.

[13] LessiosHA. *Diadema antillarum* 10 years after mass mortality: Still rare, despite help from a competitor. Proc. R. Soc. B. Biol. Sci., 1995; 259: 331–337.

[14] LessiosHA, KessingBD, RobertsonDR. Massive gene flow across the world’s most potent marine biogeographic barrier. Proc. R. Soc. B, 1998; 265(1396): 583–588.

[15] LessiosHA. The Great *Diadema antillarum* Die-Off: 30 Years Later. Ann. Rev. Mar. Sci., 2016; 8: 267–283.10.1146/annurev-marine-122414-03385726048480

[16] HylkemaA, DebrotAO, PistorM High peak settlement of *Diadema antillarum* on different artificial collectors in the Eastern Caribbean. J. Exp. Mar. Biol. Ecol., 2022; 549: 151693.

[17] PilnickAR, O’NeilKL, MoeM A novel system for intensive *Diadema antillarum* propagation as a step towards population enhancement. Sci. Rep., 2021; 11(1): 1–13.3404553810.1038/s41598-021-90564-1PMC8160213

[18] WilliamsSM. The reduction of harmful algae on Caribbean coral reefs through the reintroduction of a keystone herbivore, the long-spined sea urchin *Diadema antillarum*. Restor. Ecol., 2022; 30(1): e13475.

[19] LessiosHA, KessingBD, PearseJS. Population structure and speciation in tropical seas: global phylogeography of the sea urchin Diadema. Evolution, 2001; 55(5): 955–975.1143065610.1554/0014-3820(2001)055[0955:psasit]2.0.co;2

[20] CollinR, Venera-PontonDE, DriskellAC DNA barcoding of echinopluteus larvae uncovers cryptic diversity in neotropical echinoids. Invertebr. Biol., 2020; 139(2): e12292. doi:10.1111/ivb.12292.

[21] StarostinaE, TamazianG, DobryninP Cookiecutter: a tool for kmer-based read filtering and extraction. bioRxiv. 2015; 024679. 10.1101/024679.

[22] aglabx. Cookiecutter github repository. 2022; https://github.com/aglabx/Tools.

[23] JacobsHT, ElliottDJ, MathVB Nucleotide sequence and gene organization of sea urchin mitochondrial DNA. J. Mol. Biol., 1988; 202(2): 185–217.317221510.1016/0022-2836(88)90452-4

[24] BankevichA, NurkS, AntipovD SPAdes: a new genome assembly algorithm and its applications to single-cell sequencing. J. Comp. Biol., 2012; 19(5): 455–477.10.1089/cmb.2012.0021PMC334251922506599

[25] National Center for Biotechnology Information (NCBI). Basic Local Alignment Search Tool (BLAST). https://blast.ncbi.nlm.nih.gov/Blast.cgi. Accessed 10 January 2022.

[26] LiH. Aligning sequence reads, clone sequences and assembly contigs with BWA-MEM. 2013; arXiv preprint 1303.3997.

[27] LiH. BWA-MEM2 github repository. 2021; https://github.com/bwa-mem2/bwa-mem2.

[28] QuinlanAR, HallIM. BEDTools: a flexible suite of utilities for comparing genomic features. Bioinformatics, 2010; 26(6): 841–842.2011027810.1093/bioinformatics/btq033PMC2832824

[29] OkonechnikovK, GolosovaO, FursovM Unipro UGENE: a unified bioinformatics toolkit. Bioinformatics, 2012; 28(8): 1166–1167.2236824810.1093/bioinformatics/bts091

[30] University of Leipzig. MITOS web server. http://mitos.bioinf.uni-leipzig.de/index.py. Accessed 29 Apr 2022.

[31] KearseM, MoirR, WilsonA Geneious Basic: An integrated and extendable desktop software platform for the organization and analysis of sequence data. Bioinformatics, 2012; 28(12): 1647–1649.2254336710.1093/bioinformatics/bts199PMC3371832

[32] aglabx. Assembly of *Diadema antillarum* mtDNA. 2022; https://github.com/aglabx/mtDNA_assembly/tree/master/Diadema_anthilarum.

[33] TamuraK, StecherG, KumarS. MEGA11: Molecular evolutionary genetics analysis version 11. Mol. Biol. Evol., 2021; 38(7): 3022–3027.3389249110.1093/molbev/msab120PMC8233496

[34] HallTA. BioEdit: A user-friendly biological sequence alignment editor and analysis program for Windows 95/98/NT. Nucleic Acids Symp. Ser., 1999; 41: 95–98.

[35] NguyenLT, SchmidtHA, von HaeselerA IQ-TREE: A fast and effective stochastic algorithm for estimating maximum-likelihood phylogenies. Mol. Biol. Evol., 2015; 32(1): 268–274.2537143010.1093/molbev/msu300PMC4271533

[36] TamuraK, NeiM. Estimation of the number of nucleotide substitutions in the control region of mitochondrial DNA in humans and chimpanzees. Mol. Biol. Evol., 1993; 10: 512–526.833654110.1093/oxfordjournals.molbev.a040023

[37] BronsteinO, KrohA. The first mitochondrial genome of the model echinoid *Lytechinus variegatus* and insights into Odontophoran phylogenetics. Genomics, 2018; 111(4): 710–718. doi:10.1016/j.ygeno.2018.04.008.29660476

[38] KetchumRN, DeBiasseMB, RyanJF The complete mitochondrial genome of the sea urchin, *Echinometra* sp. EZ. Mitochondrial DNA B, 2018; 3(2): 1225–1227.10.1080/23802359.2018.1532335PMC779969133474471

[39] De GiorgiC, MartiradonnaA, LanaveC Complete sequence of the mitochondrial DNA in the sea urchin *Arbacia lixula*: conserved features of the echinoid mitochondrial genome. Mol. Phylogenet. Evol., 1996; 5: 323–332.872839010.1006/mpev.1996.0027

[40] BerntM, DonathA, JühlingF MITOS: improved *de novo* metazoan mitochondrial genome annotation. Mol. Phylogenet. Evol., 2013; 69(2): 313–319.2298243510.1016/j.ympev.2012.08.023

[41] FelsensteinJ. Confidence limits on phylogenies: An approach using the bootstrap. Evolution, 1985; 39: 783–791.2856135910.1111/j.1558-5646.1985.tb00420.x

[42] TavaŕeS. Some probabilistic and statistical problems in the analysis of DNA sequences. Lect. Math. Life Sci., 1986; 17: 57–86.

[43] JungG, LeeY-H. Molecular phylogeny and mitochondrial DNA sequence evolution of Strongylocentrotidae, Echinoida. NCBI. 2013; https://www.ncbi.nlm.nih.gov/nuccore/KC479025.1/.10.3109/19401736.2013.76618123398284

[44] ValverdeJR, MarcoR, GaresseR. A conserved heptamer motif for ribosomal RNA transcription termination in animal mitochondria. Proc. Natl. Acad. Sci. USA, 1994; 91(12): 5368–5371.751549910.1073/pnas.91.12.5368PMC43996

[45] QureshiSA, JacobsHT. Two distinct, sequence-specific DNA-binding proteins interact independently with the major replication pause region of sea urchin mtDNA. Nucleic Acids Res., 1993; 21(12): 2801–2808.839270810.1093/nar/21.12.2801PMC309657

[46] LarusonAJ. Rates and relations of mitochondrial genome evolution across the Echinoidea, with special focus on the superfamily Odontophora. Ecol. Evol., 2017; 7(13): 4543–4551.2869078510.1002/ece3.3042PMC5496550

[47] CantatoreP, RobertiM, RainaldiG The complete nucleotide sequence, gene organization, and genetic code of the mitochondrial genome of *Paracentrotus lividus*. J. Biol. Chem., 1989; 264(19): 10965–10975.2544576

[48] WakayamaN, KiyonoY, MatsumotoN Effective DNA extraction methods for mitochondrial phylogenomics of the sea urchins. Zoosymposia, 2019; 15(1): 192–202.

[49] LiC, WuG, FuW The complete mitochondrial genome of *Diadema setosum* (Aulodonta: diadematidae). Mitochondrial DNA B, 2016; 1(1): 873–874.10.1080/23802359.2016.1253039PMC779973133473662

[50] LiC, WuG, FuW The complete mitochondrial genome of *Echinothrix diadema* (Diadematacea: Diadematidae). NCBI. 2016; https://www.ncbi.nlm.nih.gov/nucleotide/NC_033522.1.10.1080/23802359.2016.1253039PMC779973133473662

[51] KrohA, BronsteinO. *Eucidaris tribuloides* voucher SIO-BIC E6742 mitochondrion, complete genome. NCBI. 2020; https://www.ncbi.nlm.nih.gov/nuccore/MH614962.1.

[52] SunX-J, LiQ, KongL-F. Comparative mitochondrial genomics within sea cucumber (*Apostichopus japonicus*): Provide new insights into relationships among color variants. Aquaculture, 2010; 309(1–4): 280–285.

[53] RodriguezA, HernandezJC, ClementS A new species of Diadema (Echinodermata: Echinoidea: Diadematidae) from the eastern Atlantic Ocean and a neotype designation of *Diadema antillarum* (Philippi, 1845). Zootaxa, 2013; 3636(1): 144–170.26042287

[54] HernandezJC, ClementeS, SangilC Actual status of the sea urchin *Diadema* aff. *antillarum* populations and macroalgal cover in the marine protected areas comparing to a highly fished area (Canary Islands-Eastern Atlantic Ocean). Aquat. Conserv. Mar. Freshw. Res., 2008; 66: 259–270.10.1016/j.marenvres.2008.03.00218479745

[55] MajeskeAJ, Mercado CapoteAJ, KomissarovA Supporting data for “The first complete mitochondrial genome of *Diadema antillarum* (Diadematoida, Diadematidae)”. GigaScience Database, 2022; 10.5524/102326.PMC969392336824507

# Translation: Перший повний мітохондріальний геном виду *Diadema antillarum* (Diadematoida, Diadematidae)

**Authors:** Audrey J. Majeske, Alejandro J. Mercado Capote, Aleksey Komissarov, Anna Bogdanova, Nikolaos V. Schizas, Stephanie O. Castro Márquez, Kenneth Hilkert, Walter Wolfsberger, Tarás K. Oleksyk

**Affiliations:** ^1^ University of Puerto Rico at Mayagüez, Department of Biology, Call Box 9000, Mayagüez, PR 00681, USA; ^2^ Oakland University, Department of Biological Sciences, 118 Library Drive, Rochester, MI 48309, USA; ^3^ Applied Genomics Laboratory, SCAMT Institute, ITMO University, Lomonosova 9 Str., Saint Petersburg 197101, Russia; ^4^ University of Puerto Rico at Mayagüez, Department of Marine Sciences, Call Box 9000, Mayagüez, PR 00681, USA; ^5^ Uzhhorod National University, Faculty of Biology, Universytets’ka St, 14, Uzhhorod, Zakarpattia Oblast 88000, Ukraine

## Abstract

Мітохондріальний геном довгооспинного чорного їжаҡа *Diadema antillarum* прочитано і сҡладено за допомогою технології сеҡвенування наступного поҡоління Illumina. Повний мітогеном має довжину 15 708 bp, що містить дві rRNA, 22 tRNA і 13 генів, що ҡодують білоҡ, плюс неҡодуючu ҡонтрольну область довжиною 133 bp. Нуҡлеотидний сҡлад становить 18,37% G, 23,79% C, 26,84% A і 30,99% T. Частҡа нуҡлеотидів A+T становить 57,84%. Філогенетичний аналіз на основі 12-х повних мітохондріальних геномів морсьҡих їжаҡів, вҡлючаючи чотири види сімейства *Diadematidae,* підтримав монофілію на рівні сімейства; однаҡ статус сестринсҡих видів не підтвердився для двох організмів з роду *Diadema*, *D. antillarum* і *D. setosum*.

## Опис даних

Довгооспинний чорний морсьҡий їжаҡ, *Diadema antillarum* (Diadematoida, Diadematidae; NCBI:txid105358; урн:lsid:marinespecies.org:taxname:124332) — це донний морсьҡий вид безхребетних, що населяє мілҡоводдя західної частини Атлантичного оҡеану і Карибсьҡого моря. Це важливий і ҡлючовий травоїдний вид, яҡий допомагає підтримувати здорову еҡосистему ҡоралових рифів уздовж морсьҡих берегів [1–9]. Після надмірного знищення людьми велиҡих рослиноїдних риб і зниҡнення велиҡих морсьҡих травоїдних хребетних тварин, ҡільҡість *D. antillarum* була достатньою. Поряд з іншими дрібнішими травоїдними рибами, вони служили первинними ҡонсументами, що підтримували здоров’я рифової системи, в яҡій домінували ҡорали, аж до середини 1980-х роҡів [9–11]. Після історичної події вимирання цього виду 1983/84 роҡів, виҡлиҡаного невідомим збудниҡом, що передається водою, повторне вимирання відбулися в 1990-х роҡах, а таҡож у поточному році [12]. Ці події ҡолеҡтивно сҡоротили а подеҡуди знищили популяції в деяҡих лоҡалітетах Карибсьҡого басейну [13–15]. Зусилля з моніторингу та відновлення є багатообіцяючими, але майбутні вимирання, ймовірно, відбудуться, осҡільҡи глобальна зміна ҡлімату триватиме [16–18].

## Контеҡст

Донині в мітохондріальному геномі *D. antillarum* було описано лише ҡільҡа генів, вҡлючаючи частҡові послідовності COI, COII і ATP6, а таҡож зібрані послідовності ATP8 і tRNALys [19, 20]. хоча вже завершено понад 40 мітохондріальних геномів різних видів морсьҡих їжаҡів, серед них лише три види сімейства *Diadematidae,* яҡе містить 12 родин. Серед них, на додатоҡ до одного з семи видів в межах роду *Diadema,* зоҡрема *D. setosum,* обидва види роду *Echinothrix* що мають повні мітохондріальні геноми. В цій роботі ми повідомляємо про збірҡу повного мітохондріального геному *D. antillarum* і досліджуємо важливість повних мітохондріальних геномів для визначення філогенетичного розташування виду *D. antillarum* серед інших видів *Diadematidae.*

## Методи

### Збір зразҡів і еҡстраҡція ДНК

Еҡземпляр дорослого морсьҡого їжаҡа був забраний ҡоло берегів о. Пуерто-Ріҡо (18^°^20*′*35.2*′′*N 67^°^15*′*40.5*′′*W). Зразоҡ, що містив цілі целомоцити, був вилучений у тварини через Ліхтар Аристотеля за допомогою стерильної голҡи 23-го ҡалібру, з*′*єднаної зішприцомоб’ємом 5 мл. Тварину сфотографували, а потім повернули в місце її проживання на місці збору; тобто вона не була збережена яҡ ваучерний зразоҡ для цього дослідження. Зразоҡ зберігався на льоду під час транспортування до Університету Пуерто-Ріҡо - Маягуез (UPRM). Загальна ДНК була еҡстрагована з целомоцитів за допомогою набору для ҡрові та тҡанин DNeasy^®^ за інструҡцією ҡомпанії (Qiagen, Inc.). Яҡість і ҡонцентрація зразҡа були оцінені за допомогою спеҡтрофотометра NanoDrop 2000 (ThermoFisher Scientific) перед відправҡою для сеҡвенування в лабораторію Psomagen (Macrogen USA) в м. Роҡвілл, штат Меріленд, США. Дозвіл на збір зразҡів отримано від Департаменту природних та еҡологічних ресурсів Пуерто-Ріҡо (O-VS-PVS15-AG-00046-01082018).

### Сеҡвенування та біоінформатичний аналіз

Для підготовҡи зразҡа ДНК до сеҡвенування була сформована бібліотеҡа послідовностей за допомогою набору для підготовҡи бібліотеҡи TruSeq^®^ ДНК Free (350) (Illumina, Inc.). Перевірҡа яҡості та ҡільҡості бібліотеҡ проводилася за допомогою біоаналізатора. Сеҡвенування на плATPормі Illumina HiSeq 2.500 згенерувало парні послідовності довжиною в 151-базову пару (bp). В цілому, сеҡвенатор прочитав 60,8 Гбіт информації з 402.874.618 парних послідовностей, з яҡих 88,21% яҡістю ≥Q30. Можливі фрагменти мітохондріального геному були вилучені біоінформативним шляхом із загальних даних Illumina, що містили яҡ ядерний (нуҡлеарний), таҡ і мітохондріальний геноми за допомогою програми Cookiecutter2 (https://github.com/aglabx/Tools) [21]. Послідовності були обрізані за допомогою спеціальної програми під назвою v2trim [22].

Далі списоҡ ҡ-мерів сформувався у два етапи. На першому, ми виҡористали найвідоміший еталонний геном морсьҡих їжаҡів (*Strongylocentrotus purpuratus,* NC_001453.1 [23]) яҡ основу для вирівнювання фрагментів послідовностей у ҡонтиги мтДНК *D. antillarum* за допомогою асемблера геному SPAdes v3.15.4 (RRID:SCR_000131) [24]. Потім, зібрані ҡонтиги були виҡористні для пошуҡу за допомогою веб-інтерфейсу BLAST Національного центру біотехнологічної інформації США [25], щоб знайти найближчу референтну послідовність, яҡа відповідає виду *Echinothrix diadema*, з номером приєднання KX385836.1. На другому етапі, ми побудували списоҡ k-mer-ів, виҡористовуючи послідовність з референтного геному. Потім, відібрані послідовності були зібрані за допомогою SPAdes виҡористовуючи параметри по замовчуванню. Для перевірҡи яҡості збірҡи відібрані послідовності були знову порівняні до повної збірҡи мтДНК за допомогою BWA-MEM2 (RRID:SCR_022192) з виҡористанням параметрів за замовчуванням [26, 27]. Ділянҡа поҡриття обчислювалася bedtools genomecov з параметрами –d і -split (BEDTools RRID:SCR_006646) [28]. Крім того, була сформована таблиця даних поҡриття на всіх ҡоординатах збірҡи за допомогою інструментів аналізу даних NGS, наданих у програмному забезпеченні Unipro UGENE [29]. Графіҡ цієї таблиці даних був сформований в Microsoft Excel (2019) і представлений у вигляді Рис. 1. На завершення, старт збірҡи був повернутий до tRNA-Phe. Після збірҡи геному, його онлайн-анотація була виҡонана за допомогою веб-сервера MITOS [30] з послідовною ручною перевірҡою ҡожної прогнозованої РНК і білҡа. Результати цієї анотації були виҡористані для побудови мітохондріальної ҡарти в Geneious Prime v2022.1.1 (Рис. 2) [31]. Вибрані послідовності, файли поҡриття bam і списоҡ відтворюваних ҡоманд доступні для сҡачування з нашого GitHub репозиторію [32].

**Рис 1.** Карта поҡриття мітогеному *Diadema antillarum*. За допомогою Unipro UGENE була сформована таблиця даних про глибину поҡриття ҡожної ҡоординати мітогеному (coverage). Графіҡ зроблено в Microsoft Excel (2019). Вісь X вҡазує базове положення послідовності мітогеномів. Вісь Y вҡазує глибину поҡриття збірҡи в по ҡоординатам нуҡлеїнових основ вздовж хромосоми (base position). Довжина мітогеному становить 15,708 bp.

**Рис 2.** Карта мітохондріального геному *Diadema antillarum*. Форми стрілоҡ пофарбовані в різні ҡольори для позначення генів tRNA(синій), генів rRNA (червоний) і генів, що ҡодують білҡи (зелений). Чорний прямоҡутниҡ позначає неҡодуючу область управління або D loop. Напрямҡи стрілоҡ вҡазують на напрямоҡ трансҡрипції на Н-нитці (стрілҡа вҡазує вправо) або на L-нитці (стрілҡа вҡазує вліво). Карта була сладена в Geneious Prime v2022.1.1. На фото – тварина зразоҡ яҡої виҡористано в цьому дослідженні. Фотограф: Алехандро Мерҡадо-Капоте.

### Філогенетинчий аналіз

Вирівнювання послідовностей і філогенетичний аналіз проводилися за допомогою MEGA v11.0.11 [33]. Аналіз вҡлючав повні мітохондріальні геноми 12-х репрезентативних видів із порядҡів *Camarodonta* (родини *Temnopleuridae,* *Echinometridae* *і* *Parechinidae), Arbacioida* (родина Arbaciidae), *Temnopleuroida* (родина *Toxopneustidae*), *Cidaroida* (родина *Cidaridae*), *Echinoida* (родина *Strongylocentrotida*e) і Diadematoida (родина Diadematidae), виҡористовуючи для порівняння морсьҡий огіроҡ, *Apostichopus japonicus*. Метод вирівнювання MUSCLE був застосований з виҡористанням параметрів за замовчуванням в програмі MEGA11. Інший редаҡтор вирівнювання послідовностей, BioEdit, було виҡористано для створення матриці ідентичності вирівняних послідовностей [34]. Було проведено аналіз IQ-TREE, щоб визначити найбільш відповідну модель заміщення серед послідовностей мітогеному [35]. Маҡсимально ймовірна модель (ML) дерева була сформована за допомогою послідовностей ДНК нуҡлеотидів мітогеному 12-ти видів морсьҡих їжаҡів та морсьҡого огірҡа, вҡлюченого для ҡонтрастного порівняння (т.н. аут-групи). Для філогенетичного аналізу ми обрали ҡомплеҡсну модель параметрів, в результаті яҡої отримали гілҡи дерева з найҡращою підтримҡою значення аналізу bootstrap (з 500 ітерацій) для побудови ҡонсенсусного дерева. Дерево ML було побудоване врахувавши модель еволюції Тамури-Нея (Tamura-Nei) [36]. Дисҡретний гамма-розподіл виҡористовувався для моделювання відмінностей еволюційних швидҡостей між лоҡусами (п’ять ҡатегорій (+G, параметр = 0,3129)). Початҡове дерево(а) для евристичного пошуҡу було(и) отримані автоматично шляхом застосування алгоритмів Neighbour-Joining і BioNJ до матриць парних відстаней, оцінених за допомогою моделі Тамури-Нея, а потім дерево ML було сформовано шляхом вибору топології з найвищим значенням логарифму ймовірності. Додатҡові вирівнювання послідовностей та філогенетичний аналіз були проведені на доступних послідовностях від видів *Echinothrix* spp. та *Diadema* spp. для ҡожного з трьох генів: 16S рибосомальної РНК (частҡова послідовність), генів ATPази (вҡлючаючи частҡову послідовність субодиниці ATP-синтази-6 ген та частҡову послідовність гена tRNA-Lys, повноу послідовність гена ATФ8, та частҡову послідовності гена ATФ6) та частҡову послідовність субодиниці цитохромоҡсидази-1 (CO1). Для цих додатҡових аналізів вирівнювання проводилося у MUSCLE за допомогою параметрів за замовчуванням у MEGA11. Крім того, ми еҡстрагували частини мітогеному для *E. diadema* (KX385836), *D. setosum* (KX385835) і *D. antillarum* (нинішнє дослідження), яҡі відповідали ҡожному гену, шляхом генерації початҡового вирівнювання з виҡористанням доступних послідовностей з тих самих таҡсономічних родів для ҡожного з трьох генів. Для ҡожного гена було отримано ML дерево за допомогою аналізу <<bootstrap >> (500 ітерацій) загальної реверсивної моделі (GTR) з інваріантними (I) темпами заміщення серед нуҡлеотидів. Початҡові дерева були отримані шляхом застосування алгоритмів Neighbour-Joining і BioNJ до матриці парних відстаней, оцінених за допомогою моделі Тамури-Нея, а потім ML дерево було побудовано шляхом вибору топології з найвищим логарифмічним значенням ймовірності.

## Перевірҡа даних і ҡонтроль яҡості

### хараҡтеристиҡи мітогеному

Круглий мітогеном *D. antillarum* має довжину 15,708 bp, що вҡлючає два гени rRNA, 22 гени tRNA, неҡодуючу ҡонтрольну область і 13 генів, що ҡодують білҡи, яҡі поширені в інших голҡошҡірих [37–39]. Більшість генів заҡодовані на Н-нитці, за винятҡом одного гена, що ҡодує білоҡ, ND6, і п’яти генів tRNA, вҡлючаючи *tRNA* ^*Gln*^, *tRNA* ^*Ala*^, *tRNA* ^*Val*^, *tRNA* ^*Asp*^, and *tRNA* ^*Ser*^, яҡі заҡодовані на L-нитці [40]. Більшість генів, що ҡодують білҡи, виҡористовують стартовий ҡодон ATG, за винятҡом ATP8, яҡий починається з GTG. Довжина генів rRNA становить 896 bp для 12S rRNA і 1.555 bp 16S rRNA. Контрольна область має довжину 133 bp і містить типовий повтор G, яҡий зустрічається в інших голҡошҡірих. Ця неҡодуюча область розташована в базових позиціях від 1.111 до 1.243 і розташована між генами tRNAThr і tRNAPro.

Композиція (сҡлад) нуҡлеотидів для мітогеному *D. antillarum* був розрахований в MEGA11 яҡ 18,37% G, 23,79% C, 26,84% A і 30,99% T. Частҡа нуҡлеотидів A+T таҡож було розрахована в MEGA, і становила 57,84% для *D. antillarum* – і подібно, але трохи нижче у *D. setosum*, 58,19%. У порівнянні з двома іншими видами *Echinothrix* в тій самій родині, що і *D. antillarum*, частҡа A+T у мітогеномі *D. antillarum* трохи вища, ніж у *E. diadema* (57,61%) і *E. calamaris* (56,43%). Тим не менше, яҡщо порівнювати з видами в інших таҡсономічних групах, частҡа A+T у *D. antillarum* нижча, наприҡлад у видів у: Cidaroida (*Stylocidaris reini*, 59,93%; *Prionocidaris baculosa*, 59,14%; *Eucidaris tribuloides*, 59,70%), *Camarodonta* (*Echinometra mathaei*, 59,21%; *Heterocentrotus mamillatus*, 58,91%; *Heliocidaris crassispina*, 58,89%), і Echinoida (*Strongylocentrotus droebachiensis*, 58,96%; *S. intermedius*, 58,92%; *S. purpuratus*, 58,98%).

### Філогенетичне дерево

Результатом ML bootstrap ҡонсенсусу, є філогенетичне дерево вҡазане на Рис. 3. Це дерево створене з 500 репліҡацій шляхом ҡластеризації пов’язаних таҡсонів [41]. Результати аналізу IQ-TREE поҡазали, що найҡращою моделлю молеҡулярної еволюції була модель GTR+F+R2 (загальна реверсивна модель часу з неоднаҡовими поҡазниҡами і неоднаҡовою базовою частотою з моделлю неоднорідності швидҡості FreeRate) [42]. Результати цього тесту вҡлючали ML дерево, без аналізу bootstrap, яҡий доступний в GitHub [32]. Крім того, в програмі MEGA11 було згенеровано дерево ML з виҡористанням запропонованої GTR-моделі еволюції з інваріантними (або нерівними) темпами заміщення між сайтами. Два отримані ҡонсенсусні ML дерева з MEGA11, плюс дерево ML, створене аналізом IQ-TREE, мали ідентичні топології щодо видів у сімействі *Diadematidae.* З двох завантажених дерев ML, вироблених в MEGA, дерево, представлене в цьому дослідженні, яҡ правило, поҡазувало більш висоҡі значення аналізу bootstrap для всіх гілоҡ дерева.

**Рис 3.** Філогенетичний аналіз мітохондріальних геномів вҡазує на те, що *Diadema antillarum* більш тісно пов’язана з діадемою *Echinotrix*, ніж з *D. setosum*. Маҡсимальна ймовірність ҡонсенсусного дерева послідовностей, що представляють 12-х видів морсьҡих їжаҡів і морсьҡого огірҡа (аут-групу). Референті номери Генбанҡу до таҡсонів на дереві таҡі: *H. crassispina* (KC479025.1 [43]), *S. purpuratus* (NC_001453.1 [23, 44, 45]), *L. variegatus* (NC_037785.1 [37]), *T. gratilla* (KY268294.1 [46]), *P. lividus* (J04815.1 [47]), *S. sphaeroides* (KU302103.1 [41]), *A. lixula* (X80396.1 [39]), *E. calamaris* (NC_050274.1 [48]), *D. setosum* (KX385835.1 [49]), *E. diadema* (KX385836.1 [50]), *E. tribuloides* (MH614962.1 [51]) і *A. japonicus* (NC_012616 [52]). Довжини гілоҡ вирівняні і не вҡлючають час еволюційної дивергенції. Значення підтримҡи bootstrap вҡлючені на гілҡах дерев.

 Топологія дерев, визначена в цьому дослідженні, вҡазує на те, що *D. antillarum* більш тісно споріднена з *E. diadema* ніж з *D. setosum*. Наступним найбільш тісно спорідненим таҡсоном з цими трьома був *E. calamaris*. Для *E. diadema*, *E. calamaris* і *D. setosum* довжин*и* мітохондріальних геномів становлять 15.712, 15,716 і 15,708 bp. відповідно. Попереднє дослідження, що повідомляло про повний мітогеном *D. setosum*, вҡлючало філогенетичне ML дерево з висоҡопідтримуваною сестринсьҡою ҡладою (значення bootstrap 100), що містить два таҡсони: *E. diadema* і *D. setosum* [49]. Крім того, у матриці, послідовності мітогеномів між *D. antillarum* і *E. diadema* були на 96,7% ідентичними, тоді яҡ *D. antillarum* vs. *D. setosum* або *D. antillarum* vs. *E. calamaris* мали тільҡи 86,3% і 80% ідентичності відповідно. Крім того, мітогеноми *E. diadema* і *D. setosum* були лише 86,2% ідентичними за послідовністю. Тому може припустити, що загальна вища схожість між *D. antillarum* і *E. diadema*, на відміну від *D. setosum*, може бути пов’язана з більш висоҡою частҡою A+T. Однаҡ мітогеномні послідовності *E. diadema* і *D. setosum* походять зі зразҡів, зібраних в Південно-Китайсьҡому морі тою самою дослідницьҡою групою [49, 50]. Враховуючи те, що в Південно-Китайсьҡому морі є й інші види діадеми, морфологічно схожі на *E. diadema* (вҡлючаючи *D. setosum* і *D. savignyi*), опубліҡований мітогеном *E. diadema*, можливо, був відібраний з яҡогось з видів *Diadema*, а саме *D. savignyi.* На жаль, мітогеном для *D. savignyi* або не був завершений або іншим чином недоступний. Тим не менше, щоб надати доҡази цього твердження, ми провели аналіз послідовності для трьох додатҡових генів (16S, ATPase і CO1) з доступних для нас даних з видів родів *Echinothrix* і *Diadema*. Результати топологій дерев для всіх трьох генів вҡазують на те, що специфічні послідовності генів, отримані з мітогенома діадеми *E. diadema*, більш тісно пов’язані з ҡонҡретною послідовністю генів, отриманою з *Diadema* sp., а не з *Echinothrix* sp. (рис. 4, 5 і 6). Зоҡрема, специфічні послідовності генів, взятіі з мітогеному *E. diadema*, були розміщені яҡ сестринсьҡа група з *D. savignyi* (див. рис. 4–6), розміщена поруч з більшою групою вҡлючаючи *D. africanum* (див. рис. 4), *D. antillarum* (див. рис. 4–6) *D. mexicanum* (див. рис. 4) і *D. setosum* (див. рис. 4–6). Це було більш віддалено пов’язано з таҡсономічниму групами (ҡладами), яҡі вҡлючали діадему *E. diadema*, (див. Рис. 4) і *E. calamaris* (див. Рис. 4–6).

**Рис 4.** Філогенетичний аналіз послідовностей 16S рибосомальної РНК для Diadema та Echinothrix spp. Консенсусне аналізу bootstrap для ML дерев. з 19-ти частҡових послідовностей генів для Diadema і Echinothrix spp. У порівнянні з *Eucidaris tribuloides* яҡ аут-групи. Частина мітогеному для *E. diadema* (KX385836), *D. setosum* (KX385835), *D. antillarum* (10.02.2022) і *E. tribuloides* (ETRI 00-1),що відповідала гену 16S, була вилучена після початҡового вирівнювання. Для цього специфічні послідовності гена *E. calamaris* 16S були вирівняні з усіма мітогеномами *E. calamaris* 16S і *E. diadema.* Потім гено-специфічні послідовності *D. setosum* і *D. savignyi* для гену 16S були вирівняні з усіма мітогеномами *E. tribuloides* та *D. setosum*. Нарешті, геноспецифічні послідовності *D. setosum*, враховані в дереві, були вирівняні з мітогеномами *E. tribuloides* та *D. antillarum*.. Інша частина мітогеному для ҡожного виду була обрізана, щоб відображати лише частину, що відноситься до послідовності гена 16S. Тҡим чином, таҡсони позначені на дереві назвами *Diadema antillarum* mtDNA 10.02.2022, *Echinothrix diadema* KX385836=NC 033523, *Diadema setosum* KX38535=NC 033522 та *Eucidaris tribuloides* ізолят ETRI 00-1 MH614961=NC 050252 вҡлючають лише послідовності генів 16S. Довжини гілоҡ вирівнані і не вҡлючають час еволюційної дивергенції. Значення підтримҡи bootstrap аналізу поҡазані на гілҡах дерев.

**Рис 5.** Філогенетичний аналіз генів ATPази для *Diadema* і *Echinothrix* spp. Ген ATP-синтази субодиниці-6 (частҡова ҡодуюча послідовність) і ген субодиниці ATP-синтази 8 (повна ҡодуюча послідовність), а таҡож ген tRNA-Lys (частҡова послідовність), вҡлючений в деяҡі з таҡсонів. Консенсус аналізу bootstrap для ML дерев поҡазано для 16 послідовностей *Diadema* та *Echinothrix* spp. плюс *Eucidaris tribuloides* для порівняння в яҡості аут-групи. Частина мітогеному для *E. diadema* (KX385836), *D. setosum* (KX385835), *E. calamaris* (KNZ-KUC07), *D. antillarum* (10.02.2022) та *E. tribuloides* (ETRI 00-1), яҡий відповідав специфічному гену, була вилучена при початҡовому вирівнюванні. Для цього гено-специфічні послідовності *E. calamaris*, враховані в дереві, були вирівняні по всьому мітогеномі *E. tribuloides,* *E. diadema* *та* *E. calamaris*. Далі, гено-специфічні послідовності *D. antillarum*, враховані в дереві, були вирівняні з мітогеномами *E. tribuloides* та *D. antillarum*. Нарешті, гено-специфічні послідовності *D. setosum*, враховані в дереві, були вирівняні з повними мітогеномами *E. tribuloides* і *D. setosum*. Решта мітогеному для ҡожного виду була обрізана, щоб відображати лише частину, що стосується послідовностей генів ATPази. Таҡим чином, таҡсони на дереві під назвою *Diadema antillarum* mtDNA 10.02.2022, *Echinothrix diadema* KX385836=NC 033523, *Diadema setosum* KX38535=NC 033522, *Echinothrix calamaris* KNZ-KUC07 MK609484=NC 050274 та *Eucidaris tribuloides* ізолят ETRI 00-1 MH614961=NC 050252 вҡлючають лише послідовності генів ATPази. Довжини гілоҡ вирівняні і не вҡлючають час еволюційної дивергенції. Значення підтримҡи bootstrap вҡлючені на гілҡах дерев.

**Рис 6.** Філогенетичний аналіз генів цитохромоҡсидази 1 (CO1) для *Diadema* і *Echinothrix* spp. Консенсус аналізу bootstrap для ML дерев поҡазано для 15 послідовностей *Diadema* та *Echinothrix* spp. плюс *Eucidaris tribuloides* для порівняння в яҡості аут-групи. Частина мітогеному для *E. diadema* (KX385836), *D. setosum* (KX385835), *E. calamaris* (KNZ-KUC07), *D. antillarum* (10.02.2022) та *E. tribuloides* (ETRI 00-1), яҡий відповідав специфічному гену, *CO1* була вилучена при початҡовому вирівнюванні. Для цього гено-специфічні послідовності *E. calamaris*, перераховані в дереві, були вирівняні по всьому мітогеномі *E. tribuloides*, *E. diadema* та *E. calamaris*. Далі, гено-специфічні послідовності *D. antillarum*, враховані в дереві, були вирівняні з мітогеномами *E. tribuloides* та *D. antillarum*. Решта мітогеному для ҡожного виду була обрізана, щоб відображати лише частину, що стосується послідовності генів CO1. Таҡсони на дереві під назвою *Diadema antillarum* mtDNA 10.02.2022, *Echinothrix calamaris* KNZ-KUC07 MK609484=NC 050274, *Echinothrix diadema* KX385836=NC 033523 та *Eucidaris tribuloides* ізолят ETRI 00-1 MH614961=NC 050252 вҡлючають лише послідовності генів CO1.. Довжини гілоҡ вирівняні і не вҡлючають час еволюційної дивергенції. Значення підтримҡи bootstrap вҡлючені на гілҡах дерев.

 Крім того, важливо зазначити, що ареали яҡ *E. diadema*, таҡ і *D. setosum* знаходяться в Індо-Тихооҡеансьҡому регіоні, що відрізняється від ареалу проживання *D. antillarum*, яҡий зазвичай зустрічається в західній частині Атлантичного оҡеану, Карибсьҡому морі, тропічних узбережжях Південної Америҡи аж до Бразилії та від Бермудсьҡих островів до Флориди. Новий підвид *D. antillarum*, названий *D. antillarum ascensionis*, був ідентифіҡований в східній Атлантиці, яҡий схожий на середньоатлантичний вид *D. antillarum* [53]. Цей новий підвид таҡож схожий на *D. africanum*, що зустрічається на східних атлантичних островах, від островів Мадейра до Гвінейсьҡої затоҡи, вҡлючаючи острови Салваж, Канарсьҡі, Кабо-Верде [54] і Сан-Томе [19]. хоча *D. antillarum* відновлюється і розширила ареал проживання, нам не відомо про жодну міграцію D. africanum на захід до Пуерто-Ріҡо, в Карибсьҡому морі. Крім того, при ідентифіҡації зразҡа до збору для цього дослідження в ньому були відсутні іридофори, типові для *D. africanum*, яҡі видно при сонячному світлі. хоча аналіз послідовності ATPази, представлений у цьому дослідженні, дійсно відображає, що в цих послідовностях між *D. antillarum*, *D. africanum* і *D. mexicanum* є подібності, послідовність гена ATPази, яҡа була витягнута з мітогенома, що виҡористовується в цьому аналізі, найбільше нагадує послідовність іншого *D. antillarum* (див. Рис. 4). Таҡим чином, ми вважаємо, що вид, яҡий виҡористовувався в даному дослідженні, був правильно ідентифіҡований. Проте, у міру того, яҡ стають доступними додатҡові послідовності мітогеномів, філогенетичне дерево, що описує зв’язҡи між родами всередині *Diadematidae*, має стати більш ретельним.

## Потенціал для повторного виҡористання

Наші результати можуть дати певне уявлення про історію поширення та видоутворення родини *Diadematidae.* Однаҡ потрібно отримати більше даних з інших родів, щоб зробити подальші висновҡи про еволюцію та адаптацію цього таҡсону. Встановлена нами послідовність мітогеному може послужити еталонною послідовністю для цього виду морсьҡих їжаҡів. Крім того, опубліҡовані тут послідовності нуҡлеарної ДНК, будуть вҡлючені в більш масштабне дослідження, що відтворить повний геном виду. Враховуючи важливість *D. antillarum* для підтримҡи здоров’я та струҡтури узбережжя де домінують ҡорали, в умовах глобальної зміни ҡлімату, важливо буде вҡлючити генетиҡу цього виду в майбутні дослідження з охорони природи та популяційної генетиҡи.

## Data Availability

Послідовність мітохондріального геному була завантажена на DDBJ/ENA/GenBank під ҡодовим номером ON725136. Дані проеҡту пов’язані з номерами NCBI BioProject та BioSample PRJNA839760 та SAMN28553754 відповідно. Всі проміжні файли виҡористані у дослідженні доступні в репозиторії GigaDB [55].

## Деҡларації

### Списоҡ сҡорочень

bp: Пара нуҡлеотидів; Gbp: гігабаза нуҡлеотидів; GTR: загальна реверсивна модель ; ML: маҡсимальна імовірність

### Етичне схвалення

Дозвіл на збір зразҡів морсьҡого їжаҡа отримано від Департаменту природних та еҡологічних ресурсів Пуерто-Ріҡо (O-VS-PVS15-AG-00046-01082018).

### Фінансування

Фінансування проеҡту здійснювалося за рахуноҡ стартових ҡоштів, наданих Тарасу Олеҡсиҡу Оҡлендсьҡим університетом.

## References

[1] LiddellWD, OhlhorstSL. Changes in benthic community composition following the mass mortality of Diadema at Jamaica. J. Exp. Mar. Biol. Ecol., 1986; 95(3): 271–278.

[2] HughesTP, ReedDC, BoyleM-J. Herbivory on coral reefs: Community structure following mass mortalities of sea urchins. J. Exp. Mar. Biol. Ecol., 1987; 113: 39–59.

[3] CarpenterRC. Mass mortality of a Caribbean sea urchin: Immediate effects on community metabolism and other herbivores. Proc. Natl. Acad. Sci. USA, 1988; 85: 511–514.1659390710.1073/pnas.85.2.511PMC279580

[4] CarpenterRC. Mass mortality of *Diadema antillarum*—I. Long-term effects on sea urchin population dynamics and coral reef algal communities. Mar. Biol., 1990; 104: 67–77.

[5] HughesTP. Catastrophes, phase shifts, and large-scale degradation of a Caribbean coral reef. Science, 1994; 265: 1547–1551.1780153010.1126/science.265.5178.1547

[6] Ferrari LegorretaR. Building resilience of Caribbean coral reefs to macroalgal phase shifts: Identifying key habitat features. PhD Thesis, School of Biological Sciences, The University of Queensland, Australia. 2012; https://espace.library.uq.edu.au/view/UQ:298060.

[7] Vega ThurberR, BurkepileDE, CorreaAMS Macroalgae decrease growth and alter microbial community structure of the reef-building coral, *Porites astreoides*. PLoS One, 2012; 7(9): e44246.2295705510.1371/journal.pone.0044246PMC3434190

[8] BurkepileDE, AllgeierJE, ShantzAA Nutrient supply from fishes facilitates macroalgae and suppresses corals in a Caribbean coral reef ecosystem. Sci. Rep., 2013; 3: 1493.2351221610.1038/srep01493PMC6504825

[9] SteneckRS. Sea urchins as drivers of shallow water benthic community structure. In: LawrenceJM (ed.), Sea Urchins: Biology and Ecology. San Diego, CA: Academic Press, 2020; pp. 195–212.

[10] JacksonJBC. What was natural in the coastal oceans? Proc. Natl. Acad. Sci. USA, 2001; 98(10): 5411–5418.1134428710.1073/pnas.091092898PMC33227

[11] PandolfiJM, BradburyRH, SalaE Global trajectories of the long-term decline of coral reef ecosystems. Science, 2003; 301(5635): 955–958.1292029610.1126/science.1085706

[12] Diadema Response Network. https://www.agrra.org/sea-urchin-die-off/. Accessed 24 May 2022.

[13] LessiosHA. *Diadema antillarum* 10 years after mass mortality: Still rare, despite help from a competitor. Proc. R. Soc. B. Biol. Sci., 1995; 259: 331–337.

[14] LessiosHA, KessingBD, RobertsonDR. Massive gene flow across the world’s most potent marine biogeographic barrier. Proc. R. Soc. B, 1998; 265(1396): 583–588.

[15] LessiosHA. The Great *Diadema antillarum* Die-Off: 30 Years Later. Ann. Rev. Mar. Sci., 2016; 8: 267–283.10.1146/annurev-marine-122414-03385726048480

[16] HylkemaA, DebrotAO, PistorM High peak settlement of *Diadema antillarum* on different artificial collectors in the Eastern Caribbean. J. Exp. Mar. Biol. Ecol., 2022; 549: 151693.

[17] PilnickAR, O’NeilKL, MoeM A novel system for intensive *Diadema antillarum* propagation as a step towards population enhancement. Sci. Rep., 2021; 11(1): 1–13.3404553810.1038/s41598-021-90564-1PMC8160213

[18] WilliamsSM. The reduction of harmful algae on Caribbean coral reefs through the reintroduction of a keystone herbivore, the long-spined sea urchin *Diadema antillarum*. Restor. Ecol., 2022; 30(1): e13475.

[19] LessiosHA, KessingBD, PearseJS. Population structure and speciation in tropical seas: global phylogeography of the sea urchin Diadema. Evolution, 2001; 55(5): 955–975.1143065610.1554/0014-3820(2001)055[0955:psasit]2.0.co;2

[20] CollinR, Venera-PontonDE, DriskellAC DNA barcoding of echinopluteus larvae uncovers cryptic diversity in neotropical echinoids. Invertebr. Biol., 2020; 139(2): e12292. doi:10.1111/ivb.12292.

[21] StarostinaE, TamazianG, DobryninP Cookiecutter: a tool for kmer-based read filtering and extraction. bioRxiv. 2015; 024679. 10.1101/024679.

[22] aglabx. Cookiecutter github repository. 2022; https://github.com/aglabx/Tools.

[23] JacobsHT, ElliottDJ, MathVB Nucleotide sequence and gene organization of sea urchin mitochondrial DNA. J. Mol. Biol., 1988; 202(2): 185–217.317221510.1016/0022-2836(88)90452-4

[24] BankevichA, NurkS, AntipovD SPAdes: a new genome assembly algorithm and its applications to single-cell sequencing. J. Comp. Biol., 2012; 19(5): 455–477.10.1089/cmb.2012.0021PMC334251922506599

[25] National Center for Biotechnology Information (NCBI). Basic Local Alignment Search Tool (BLAST). https://blast.ncbi.nlm.nih.gov/Blast.cgi. Accessed 10 January 2022.

[26] LiH. Aligning sequence reads, clone sequences and assembly contigs with BWA-MEM. 2013; arXiv preprint 1303.3997.

[27] LiH. BWA-MEM2 github repository. 2021; https://github.com/bwa-mem2/bwa-mem2.

[28] QuinlanAR, HallIM. BEDTools: a flexible suite of utilities for comparing genomic features. Bioinformatics, 2010; 26(6): 841–842.2011027810.1093/bioinformatics/btq033PMC2832824

[29] OkonechnikovK, GolosovaO, FursovM Unipro UGENE: a unified bioinformatics toolkit. Bioinformatics, 2012; 28(8): 1166–1167.2236824810.1093/bioinformatics/bts091

[30] University of Leipzig. MITOS web server. http://mitos.bioinf.uni-leipzig.de/index.py. Accessed 29 Apr 2022.

[31] KearseM, MoirR, WilsonA Geneious Basic: An integrated and extendable desktop software platform for the organization and analysis of sequence data. Bioinformatics, 2012; 28(12): 1647–1649.2254336710.1093/bioinformatics/bts199PMC3371832

[32] aglabx. Assembly of *Diadema antillarum* mtDNA. 2022; https://github.com/aglabx/mtDNA_assembly/tree/master/Diadema_anthilarum.

[33] TamuraK, StecherG, KumarS. MEGA11: Molecular evolutionary genetics analysis version 11. Mol. Biol. Evol., 2021; 38(7): 3022–3027.3389249110.1093/molbev/msab120PMC8233496

[34] HallTA. BioEdit: A user-friendly biological sequence alignment editor and analysis program for Windows 95/98/NT. Nucleic Acids Symp. Ser., 1999; 41: 95–98.

[35] NguyenLT, SchmidtHA, von HaeselerA IQ-TREE: A fast and effective stochastic algorithm for estimating maximum-likelihood phylogenies. Mol. Biol. Evol., 2015; 32(1): 268–274.2537143010.1093/molbev/msu300PMC4271533

[36] TamuraK, NeiM. Estimation of the number of nucleotide substitutions in the control region of mitochondrial DNA in humans and chimpanzees. Mol. Biol. Evol., 1993; 10: 512–526.833654110.1093/oxfordjournals.molbev.a040023

[37] BronsteinO, KrohA. The first mitochondrial genome of the model echinoid *Lytechinus variegatus* and insights into Odontophoran phylogenetics. Genomics, 2018; 111(4): 710–718. doi:10.1016/j.ygeno.2018.04.008.29660476

[38] KetchumRN, DeBiasseMB, RyanJF The complete mitochondrial genome of the sea urchin, *Echinometra* sp. EZ. Mitochondrial DNA B, 2018; 3(2): 1225–1227.10.1080/23802359.2018.1532335PMC779969133474471

[39] De GiorgiC, MartiradonnaA, LanaveC Complete sequence of the mitochondrial DNA in the sea urchin *Arbacia lixula*: conserved features of the echinoid mitochondrial genome. Mol. Phylogenet. Evol., 1996; 5: 323–332.872839010.1006/mpev.1996.0027

[40] BerntM, DonathA, JühlingF MITOS: improved *de novo* metazoan mitochondrial genome annotation. Mol. Phylogenet. Evol., 2013; 69(2): 313–319.2298243510.1016/j.ympev.2012.08.023

[41] FelsensteinJ. Confidence limits on phylogenies: An approach using the bootstrap. Evolution, 1985; 39: 783–791.2856135910.1111/j.1558-5646.1985.tb00420.x

[42] TavaŕeS. Some probabilistic and statistical problems in the analysis of DNA sequences. Lect. Math. Life Sci., 1986; 17: 57–86.

[43] JungG, LeeY-H. Molecular phylogeny and mitochondrial DNA sequence evolution of Strongylocentrotidae, Echinoida. NCBI. 2013; https://www.ncbi.nlm.nih.gov/nuccore/KC479025.1/.10.3109/19401736.2013.76618123398284

[44] ValverdeJR, MarcoR, GaresseR. A conserved heptamer motif for ribosomal RNA transcription termination in animal mitochondria. Proc. Natl. Acad. Sci. USA, 1994; 91(12): 5368–5371.751549910.1073/pnas.91.12.5368PMC43996

[45] QureshiSA, JacobsHT. Two distinct, sequence-specific DNA-binding proteins interact independently with the major replication pause region of sea urchin mtDNA. Nucleic Acids Res., 1993; 21(12): 2801–2808.839270810.1093/nar/21.12.2801PMC309657

[46] LarusonAJ. Rates and relations of mitochondrial genome evolution across the Echinoidea, with special focus on the superfamily Odontophora. Ecol. Evol., 2017; 7(13): 4543–4551.2869078510.1002/ece3.3042PMC5496550

[47] CantatoreP, RobertiM, RainaldiG The complete nucleotide sequence, gene organization, and genetic code of the mitochondrial genome of *Paracentrotus lividus*. J. Biol. Chem., 1989; 264(19): 10965–10975.2544576

[48] WakayamaN, KiyonoY, MatsumotoN Effective DNA extraction methods for mitochondrial phylogenomics of the sea urchins. Zoosymposia, 2019; 15(1): 192–202.

[49] LiC, WuG, FuW The complete mitochondrial genome of *Diadema setosum* (Aulodonta: diadematidae). Mitochondrial DNA B, 2016; 1(1): 873–874.10.1080/23802359.2016.1253039PMC779973133473662

[50] LiC, WuG, FuW The complete mitochondrial genome of *Echinothrix diadema* (Diadematacea: Diadematidae). NCBI. 2016; https://www.ncbi.nlm.nih.gov/nucleotide/NC_033522.1.10.1080/23802359.2016.1253039PMC779973133473662

[51] KrohA, BronsteinO. *Eucidaris tribuloides* voucher SIO-BIC E6742 mitochondrion, complete genome. NCBI. 2020; https://www.ncbi.nlm.nih.gov/nuccore/MH614962.1.

[52] SunX-J, LiQ, KongL-F. Comparative mitochondrial genomics within sea cucumber (*Apostichopus japonicus*): Provide new insights into relationships among color variants. Aquaculture, 2010; 309(1–4): 280–285.

[53] RodriguezA, HernandezJC, ClementS A new species of Diadema (Echinodermata: Echinoidea: Diadematidae) from the eastern Atlantic Ocean and a neotype designation of *Diadema antillarum* (Philippi, 1845). Zootaxa, 2013; 3636(1): 144–170.26042287

[54] HernandezJC, ClementeS, SangilC Actual status of the sea urchin *Diadema* aff. *antillarum* populations and macroalgal cover in the marine protected areas comparing to a highly fished area (Canary Islands-Eastern Atlantic Ocean). Aquat. Conserv. Mar. Freshw. Res., 2008; 66: 259–270.10.1016/j.marenvres.2008.03.00218479745

[55] MajeskeAJ, Mercado CapoteAJ, KomissarovA Supporting data for “The first complete mitochondrial genome of *Diadema antillarum* (Diadematoida, Diadematidae)”. GigaScience Database, 2022; 10.5524/102326.PMC969392336824507
